# A Deep Learning-Based Sentiment Classification Model for Real Online Consumption

**DOI:** 10.3389/fpsyg.2022.886982

**Published:** 2022-04-14

**Authors:** Yang Su, Yan Shen

**Affiliations:** ^1^School of Art, Anhui Polytechnic University, Wuhu, China; ^2^Ideological, Political and Basic Teaching Department, Communication University of China, Nanjing, China

**Keywords:** consumer sentiment, product reviews, sentiment analysis, long-and short-term memory networks, feature mapping

## Abstract

Most e-commerce platforms allow consumers to post product reviews, causing more and more consumers to get into the habit of reading reviews before they buy. These online reviews serve as an emotional feedback of consumers’ product experience and contain a lot of important information, but inevitably there are malicious or irrelevant reviews. It is especially important to discover and identify the real sentiment tendency in online reviews in a timely manner. Therefore, a deep learning-based real online consumer sentiment classification model is proposed. First, the mapping relationship between online reviews of goods and sentiment features is established based on expert knowledge and using fuzzy mathematics, thus mapping the high-dimensional original text data into a continuous low-dimensional space. Secondly, after obtaining local contextual features using convolutional operations, the long-term dependencies between features are fully considered by a bidirectional long- and short-term memory network. Then, the degree of contribution of different words to the text is considered by introducing an attention mechanism, and a regular term constraint is introduced in the objective function. The experimental results show that the proposed convolutional attention–long and short-term memory network (CA–LSTM) model has a higher test accuracy of 83.3% compared with other models, indicating that the model has better classification performance.

## Introduction

With the rapid growth of the Internet and large e-commerce websites (e.g., Jingdong, Tmall, Taobao, Amazon, etc.), online shopping has become increasingly popular. In China, the size of online shopping users reached 569 million in 2018. Consumers can use e-commerce websites to freely express their experiences and feelings about the products they buy, and a 2014 study by Ariadne Consulting reported that about 63.9% of online consumers write product reviews after shopping (Cambria et al., 2018; Capraro and Vanzo, 2018; Dragoni et al., 2018; Mäntylä et al., 2018). In total, 72.6% of consumers read other consumers’ product reviews before purchasing a product to make quicker and easier decisions. This review information is of great value to both the individual consumer and the company. Therefore, the analysis of sentiment tendency of reviews becomes particularly important.

The presence of a large number of online watermen has led to the existence of more than 15% of false online reviews on e-commerce websites. The presence of fake reviews can convey false emotional tendencies to consumers, leading to user attrition and even to wrong decision-making behavior (Alkubaisi et al., 2018; Wei et al., 2018; Samah, 2021; Shamrat et al., 2021). In addition, managers of e-commerce websites analyze the sentiments reflected in consumer reviews in order to rate merchants and thus eventually lower the ranking of merchants with bad reviews. Therefore, it is particularly important to detect and accurately identify the true sentiment tendencies in online reviews in a timely manner.

Obtaining the sentiment tendency of a review by hand can have many problems in terms of objectivity, accuracy, and efficiency. Therefore, how to use natural language processing and artificial intelligence technologies to automatically and efficiently mine the sentiment tendency of review texts has become a popular and highly significant research topic today (Dehkharghani et al., 2014; Ghiassi and Lee, 2018; Weng et al., 2018; Liu et al., 2021; Naeem et al., 2021). The traditional rule-based text sentiment analysis methods mainly analyze the sentiment of texts based on sentiment dictionaries, feature statistics and sentiment templates obtained from human experience or expert opinions. Therefore, the traditional rule-based methods cannot distinguish the false sentiment from the real sentiment in the review text.

Therefore, this paper aims to investigate the problem of identifying the true affective tendencies of reviews in large e-commerce websites, and to determine whether consumers’ true affective tendencies are positive or negative by establishing a mapping relationship between online reviews of products and affective characteristics. Based on long and short-term memory network (LSTM), a classification model is constructed by extracting real consumer sentiment feature indicators from the context of reviews with the help of attention mechanisms and convolution operations, with the aim of reducing the proportion of spam reviews and helping to improve the overall review quality of the website.

The rest of the paper is organized as follows: In section “Related Research,” a related research is studied in detail, while section “The Connection Between Product Reviews and Emotional Characteristics” provides the detailed connection between product reviews and sentimental characteristics. Section “Real Online Consumer Sentiment Classification Model” provides detailed real online consumer sentiment classification model. Section “Experiment and Result Analysis” provides the results and discussion. Finally, the paper is concluded in section “Conclusion.”

## Related Research

Fake reviews, also known as Deceptive review, are divided into three main categories (Chen et al., 2015; Hou, 2020; Khan et al., 2020): (1) Untruthful opinion, i.e., exaggerated praise or excessive denigration of products or services, thus influencing users’ opinions or consumption behavior; (2) Reviews on brands only) i.e., the review content is not related to the product itself, but mainly evaluates the brand, manufacturer or seller of the product, etc.; (3) Non-reviews, i.e., users post non-repetitive reviews, advertisements or links, etc., that are not related to the target product.

The core of online review mining mainly lies in the judgment of sentiment tendency. Currently, in the research of identifying spam reviews, most researchers use text sentiment analysis to mine the sentiment tendency of user reviews and analyze users’ recognition of products or services. For example, Zhang et al. (2020) proposed a logical model for evaluating the sentiment tendency based on review product attributes, starting from the perspective of online product review attribute sentiment. The three key dictionaries in this model are generic attribute dictionary, personalized attribute dictionary, and sentiment tendency dictionary. Rehman et al. (2017) proposed a set of rules to identify spam reviews and combined this rule with a time series approach to identify the sentiment polarity and sentiment intensity of spam reviews. In addition, some other researchers have utilized sentiment analysis techniques in extracting feature metrics. For example, Tao and Liu (2018) proposed a novel detection framework called novel detection framework to mine sentiment features for potential pairwise features in a large number of comment texts to model the relationship between spam comments more robustly.

In the research of identifying spam reviews, besides the key steps of clarifying the concept of spam reviews and mining the features of reviews, the most important is to build a text classification model. At present, the more commonly used methods of classification models are mainly divided into three types: (1) Supervised machine learning methods, Semi-supervised learning methods and Unsupervised learning methods. Liu (2015) proposed a method to detect spam comments using Stylometric features and construct a text classification model using Support Vector Machine (SVM) and plain Bayesian classification algorithm. Suhasini and Badugu (2018) provided an unsupervised recognition model of basic term association knowledge to help improve the efficiency of mining potential classification features.

For the problem that existing online review sentiment classification methods do not distinguish between true and false sentiment features, a deep learning-based sentiment classification model for true online consumption is proposed. The experimental results show that the proposed convolutional attention LSTM (CA-LSTM) model has higher testing accuracy compared with other models, which verifies the advancement and effectiveness of the model. Based on the bidirectional LSTM, the classification model is constructed by extracting real consumer sentiment feature indicators from the context of reviews with the help of attention mechanism and convolution operation.

The main innovation points of this paper are as follows:

(1) To solve the problem that traditional mathematical methods are difficult to analyze sentiment features directly and quantitatively, we establish the mapping relationship between product online reviews and sentiment features with the help of expert knowledge and using fuzzy mathematics, so as to clarify the relationship between consumers’ real psychology and review attributes using a spatial Boolean model.
(2) In order to effectively identify the relationship matrix between product attributes and sentiment features, the classification model is constructed by extracting real consumer sentiment feature indicators from the context of reviews with the help of attention mechanism and convolution operation on the basis of Bi-directional short-term memory network, which improves the correct rate of review text classification.

## The Connection Between Product Reviews and Emotional Characteristics

Consumers usually assess the emotional characteristics conveyed by a product based on their own experiences, personal preferences, etc. during the process of purchasing the product online. It can be seen that the true sentiment characteristics of product reviews are usually expressed through the review attributes. Therefore, in order to identify the real emotional tendencies of consumers, it is first necessary to link the product review model and the emotional features, i.e., to establish the relationship between product reviews and emotional features, so as to extract the emotional features of the reviews based on the review attributes. In this paper, the link between review attributes and sentiment features is established with the help of expert knowledge and using fuzzy mathematics.

### Fuzzy Mathematics Is Introduced

Fuzzy mathematics is a branch of mathematics that studies and deals with fuzzy phenomena and can effectively deal with concepts that are more subjective. For this reason, this paper introduces the theory of fuzzy mathematics (Kang et al., 2018) and adopts the method of fuzzy linguistic variables to describe the emotional characteristics of users in the process of consumption and quantify the qualitative and subjective information of users.

**FIGURE 1:**
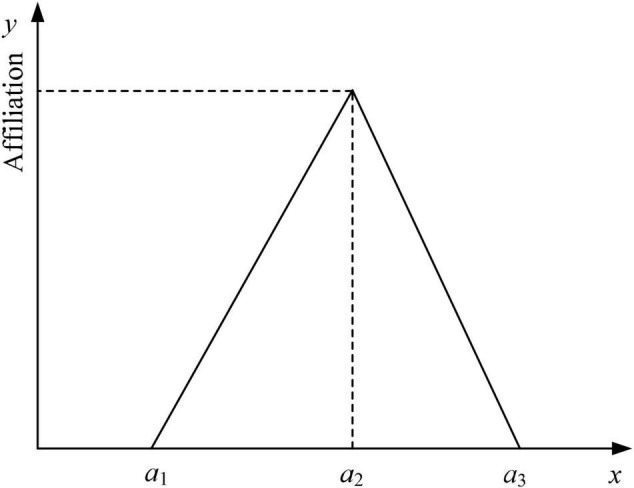
Membership function of triangle fuzzy number.

Linguistic variables are usually represented by fuzzy mathematical methods, which means that they are converted into fuzzy numbers for quantification purposes. Because of the simplicity of the triangular affiliation function representation and the ease of describing emotional needs, in this study, triangular fuzzy numbers are used to represent linguistic variables. Assuming that $\tilde{a}$ is a triangular fuzzy number, represented by a set of ternary numbers as $\tilde{a}=\left(a_{1},a_{2},a_{3}\right)$, where *a*_1_ < *a*_2_ < *a*_3_, the definition of the triangular affiliation function $\mu_{\tilde{a}}\,\left(x\right)$ is shown in Equation (1), and the corresponding affiliation function graph is given in Figure 1.


(1) $$\mu_{\tilde{a}}\,\left(x\right)=\left\{ \begin{array}{cc} \frac{x-a_{1}}{a_{2}-a_{1}} & a_{1}\leq x<a_{2}\\ \frac{a_{3}-x}{a_{3}-a_{2}} & a_{2}\leq x<a_{3}\\ 0 & \text{others} \end{array}\right.$$

**FIGURE 2:**
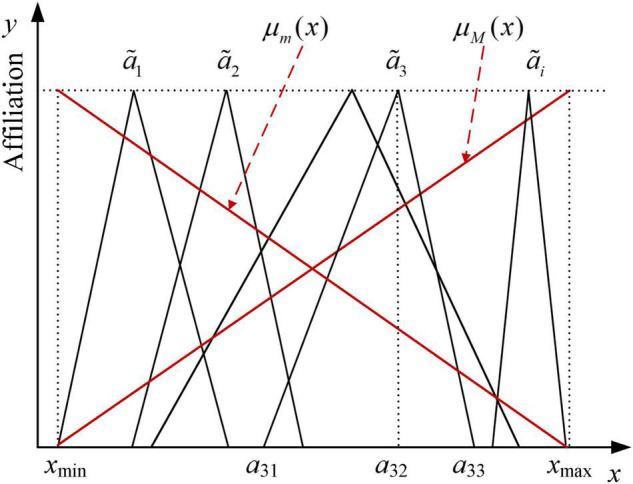
Affiliation function of Maximizing-minimizing set.

It is important to note that fuzzy numbers are not explicit values and are difficult to compare directly. In order to accomplish this, it is necessary to defuzzify the triangular fuzzy numbers, i.e., convert the fuzzy sets into the corresponding single values. Commonly used methods include the prime method, the maximum subordination method, and the Maximum-minimum set method. Suppose ${\tilde{a}}_{1},{\tilde{a}}_{2},\cdots ,{\tilde{a}}_{n}$ are *n* triangular fuzzy numbers, where ${\tilde{a}}_{i}=\left(a_{i\,1},a_{i\,2},a_{i\,3}\right)$, then as shown in Figure 2, the affiliation functions corresponding to the maximum set M and the minimum set m are shown in Equations (2) and (3), respectively.


(2) $$\mu_{M}\,\left(x\right)=\left\{ \begin{array}{cc} \frac{x-x_{\min}}{x_{\max}-x_{\min}} & x_{\min}\leq x\leq x_{\max}\\ 0 & \text{others} \end{array}\right.$$

(3) $$\mu_{m}\,\left(x\right)=\left\{ \begin{array}{cc} \frac{x-x_{\max}}{x_{\min}-x_{\max}} & x_{\min}\leq x\leq x_{\max}\\ 0 & \text{others} \end{array}\right.$$

### Representation of the Review Model

In order to quantify the sentiment characteristics embedded in the reviews, it is necessary to represent the review attributes by means of a mathematical model (Kakad and Dhage, 2021). Reviews generally contain multiple attributes, each of which in turn includes a varying number of attribute values. Assume that the review has *t* attributes, the *j*-th attribute contains *c*_*j*_ attribute values, and *f*_*i*_(*j* → *k*)denotes the *k-*th attribute value of the *j*-th attribute of item *i*, where *j* = 1, 2, ⋯, *t*; *k* = 1, 2, ⋯, *c*_*j*_. For the same review, each attribute will and will only be represented as one of the attribute values, i.e., the constraints are as follows:


(4) $$\sum_{k=1}^{c_{j}}f_{i}\left(j\to k\right)=1$$

In this paper, the different attribute values exhibited by the comment attributes are used to distinguish and describe the comments, and the spatial Boolean model representation is applied, then the comment model can be expressed in the following form.


(5) $$F_{i}=\left(f_{i}\left(1\to 1\right),f_{i}\left(1\to 2\right),\cdots f_{i}\left(t\to c_{t}\right)\right)$$

### Semantic Ambiguity of Sentiment Features

**FIGURE 3:**
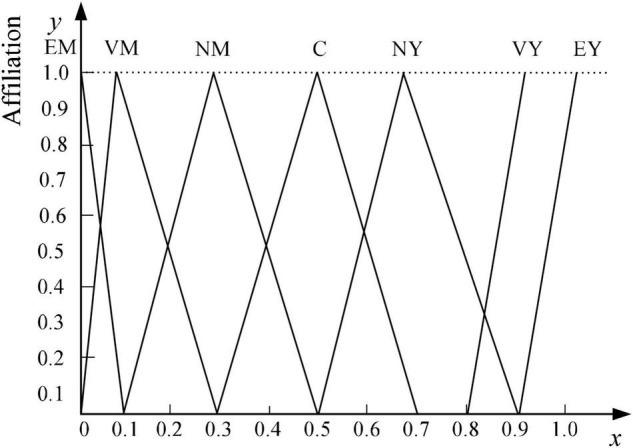
Affiliation functions of linguistic variables describing users’ emotional characteristics.

There are many fuzzy factors when using sentiment vocabulary to describe the sentiment characteristics of reviews, and the concept itself has certain uncertainty, which is difficult to analyze directly and quantitatively using traditional mathematical methods. Therefore, this paper introduces linguistic variables and combines fuzzy mathematical methods to assign numerical values to fuzzy subjective information to complete the semantic fuzzification of sentiment features. In this paper, a seven-level evaluation scale (Lanjewar et al., 2015) is used to describe the affective characteristics of reviews, and the specific linguistic variable values and triangular fuzzy numbers are shown in Table 1, and the corresponding affiliation functions are shown in Figure 3, taking clothing product reviews as an example.

**TABLE 1:** Linguistic variables for affective characterization.

Linguistic variable values	Triangular fuzzy number
Extreme matur (EM)	(0.0, 0.0, 0.1)
Very mature (VM)	(0.0, 0.1, 0.3)
Normal mature (NM)	(0.1, 0.3, 0.5)
Central (C)	(0.3, 0.5, 0.7)
Normal young (NY)	(0.5, 0.7, 0.9)
Very young (VY)	(0.7, 0.9, 1.0)
Extreme young (EY)	(0.9, 1.0, 1.0)

### The Relationship Between Review Attributes and Sentiment Characteristics

In order to establish the relationship between review attributes and emotional characteristics, this paper combines qualitative and quantitative methods to transform domain expert knowledge into quantified data. According to the relationship between linguistic variables and triangular fuzzy numbers in Table 1, the evaluation results are expressed in the form of triangular fuzzy numbers, such as $\tilde{r}=\left(a,b,c\right)$, then the evaluation results of three experts can be expressed as ${\tilde{r}}_{l\,\left(1\right)}^{j\to k}$, ${\tilde{r}}_{l\,\left(2\right)}^{j\to k}$, ${\tilde{r}}_{l\,\left(3\right)}^{j\to k}$, respectively. For the weighted average of expert knowledge, then the relationship between sentiment features and comment attributes can be calculated by Equation (6).


(6) $${\tilde{r}}_{l}^{j\to k}=\frac{1}{3}\,\left[⊕{\tilde{r}}_{l\,\left(1\right)}^{j\to k}⊕{\tilde{r}}_{l\,\left(2\right)}^{j\to k}⊕{\tilde{r}}_{l\,\left(3\right)}^{j\to k}\right]$$

where *l* denotes the sentiment feature, *j* denotes the comment attribute, and *j* → *k* denotes the attribute value of attribute *j*.

To facilitate the subsequent analytical calculations, the correspondence between the above-mentioned comment attributes and emotional characteristics is expressed in the form of Equation (7).


(7) $$U=\left(\begin{array}{cccc} u_{T}\,\left({\tilde{r}}_{1}^{1\to 1}\right) & u_{T}\,\left({\tilde{r}}_{2}^{1\to 1}\right) & \ldots & u_{T}\,\left({\tilde{r}}_{n}^{1\to 1}\right)\\ u_{T}\,\left({\tilde{r}}_{1}^{1\to 2}\right) & u_{T}\,\left({\tilde{r}}_{2}^{1\to 2}\right) & \ldots & u_{T}\,\left({\tilde{r}}_{n}^{1\to 2}\right)\\ \vdots & \vdots & u_{T}\,\left({\tilde{r}}_{l}^{j\to k}\right) & \vdots \\ u_{T}\,\left({\tilde{r}}_{1}^{t\to c_{t}}\right) & u_{T}\,\left({\tilde{r}}_{2}^{t\to c_{t}}\right) & \ldots & u_{T}\,\left({\tilde{r}}_{n}^{t\to c_{t}}\right) \end{array}\right)$$

where $u_{T}\,\left({\tilde{r}}_{l}^{j\to k}\right)$ denotes the correspondence between attribute value *j* → *k* and sentiment feature *i*.

## Real Online Consumer Sentiment Classification Model

### The Proposed Convolutional Attention–Long and Short-Term Memory Network Model

**FIGURE 4:**
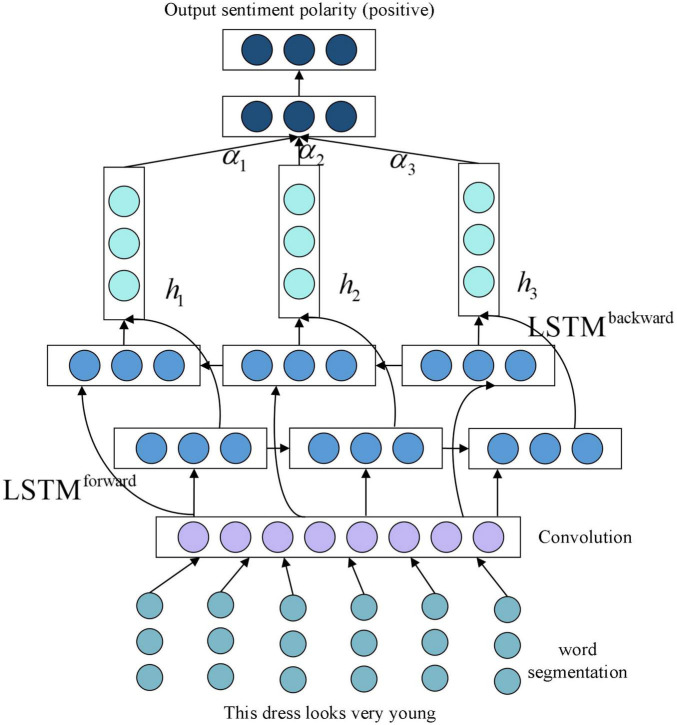
Convolutional attention-long and short-term memory network (CA-LSTM) model.

In order to solve the problem of real sentiment analysis of product reviews, this paper proposes a CA-LSTM model based on typical LSTMs in deep learning techniques, as shown in Figure 4. We regard false comments as junk comments, and use fuzzy mathematics to establish the relationship matrix between commodity attributes and emotional characteristics, thus eliminating the influence of these false comments. Note that the input of CA-LSTM model is the above relational matrix. This model consists of the following five main steps.

Step 1: text representation using the relationship matrix of comment attributes and sentiment features.

Step 2: Obtaining local features using convolution operations.

Step 3: taking full account of the long-term dependencies between features using the BiLSTM model.

Step 4: introduction of an attention mechanism to represent the veracity of different features.

Step 5: Sentiment classification using classifier.

(1) Input layer.

The relationship matrix **U** of comment attributes and sentiment features is used to vectorize the unlogged words for initialization. A comment in the input layer can be vectorized as follows:


(8) $${\text{x}}_{1:n}=\text{U}\,\left({\text{x}}_{1}⊕{\text{x}}_{2}⊕\ldots ⊕{\text{x}}_{n}\right)$$

where *n* is the length of a comment.

(2) Convolution layer.

The role of the convolution kernel is to get the local features of the input data by window sliding. The convolution filter *m* ∈ R^*h*×*k*^ is a convolution operation on a *k*-dimensional word vector with window *h*. The new feature of the *i*-th word in a comment is represented as follows:


(9) $$c_{i}=f\,\left(\text{m}\cdot {\text{x}}_{i:i+h-1}+\text{b}\right)$$

where **m** and **b** are the weight matrix and bias, respectively. *f* is the non-linear activation function Relu.

The characteristics of the comments are expressed as follows:


(10) $$\text{C}=\left[c_{1},c_{2},\cdots ,c_{n-h+1}\right],\text{C}\in R^{n-h+1}$$

(3) Fully connected layer.

The output *y_i_* of this layer is connected by each feature mapping, which can be expressed as follows:


(11) $${\text{y}}_{i}={\text{C}}_{1}^{i}⊕{\text{C}}_{2}^{i}⊕\cdots ⊕{\text{C}}_{p}^{i}$$

(4) Bidirectional LSTM layer.

Although LSTM can solve the problem of long-term dependency, it does not utilize the contextual information of the text, so this paper adopts the Bidirectional LSTM (BiLSTM) model (Contractor et al., 2021; Li et al., 2021; Liu and Zhang, 2021) to consider the contextual information of the text at the same time. The forward LSTM and backward LSTM can obtain the above and below information of the input sequence, respectively, by two LSTMs to get the hidden layer states with opposite timing and connect them to get the same output. The BiLSTM model can effectively improve the accuracy. The hidden state of BiLSTM at time *t* contains forward ${\text{h}}_{t}^{\text{forward}}$ and backward ${\text{h}}_{t}^{\text{backward}}$.


(12) $${\text{h}}_{t}^{\text{forward}}={\text{LSTM}}^{\text{forward }}\,\left({\text{h}}_{t-1},{\text{x}}_{t},{\text{c}}_{t-1}\right),t\in \left[1,T\right]$$

(13) $${\text{h}}_{t}^{\text{backward}}={\text{LSTM}}^{\text{backward }}\,\left({\text{h}}_{t-1},{\text{x}}_{t},{\text{c}}_{t-1}\right),t\in \left[T,1\right]$$

(14) $${\text{H}}_{t}=\left[{\text{h}}_{t}^{\text{forward}},{\text{h}}_{t}^{\text{backward}}\right]$$

The output *H*_*t*_ of the BiLSTM is used as the feature vector of the text.

### Attention Mechanism

For the sentiment classification task, the sentiment words in a sentence have a very critical role in discriminating the sentiment tendency of the whole sentence. Therefore, the CA-LSTM model is proposed to calculate the attention weight of each word in the text by introducing an attention mechanism, so that the hidden state at the moment of the sentiment word has a greater contribution to the sentiment classification.

In the CA-LSTM model, firstly, the attention weight occupied by the hidden state *H*_*t*_ at each moment is denoted as α_*t*_, and then, the hidden state *v* for classification is obtained by weighted accumulation, which can be expressed as follows:


(15) $${\text{u}}_{t}=\tanh\left({\text{W}}^{a\,t\,t}\,{\text{H}}_{t}+{\text{b}}^{a\,t\,t}\right)$$

(16) $$\alpha_{t}=\frac{\exp\left({\text{u}}_{t}^{\text{T}}\,{\text{u}}_{w}\right)}{\sum_{t}\exp\left({\text{u}}_{t}^{\text{T}}\,{\text{u}}_{w}\right)}$$

(17) $$\text{v}=\sum_{t}\alpha_{t}\,{\text{H}}_{t}$$

where **u**_*t*_ is the hidden cell state of **H**_*t*_, **u**_*w*_ is the context vector, and both **W**^*att*^ and **b**^*att*^ are the attention mechanism parameters.

Finally, the attention mechanism output *v* is input to the Softmax function for sentiment classification, and the classification results are as follows:


(18) $$\hat{y}=s\,o\,f\,t\,m\,a\,x\,\left({\text{w}}_{s}\,\text{v}+{\text{b}}_{s}\right)$$

where **w**_*s*_ and **b**_*s*_ are the weight matrix and bias, respectively.

### Model Training

The parameters of the model are updated by Adam optimization algorithm and small batch strategy, and cross-entropy loss is used as the loss function of the sentiment classifier. The cross-entropy loss function is formulated as follows:


(19) $$L_{\text{sentiment}}=-\sum_{i}y_{i}\,\log{\hat{y}}_{i}+\lambda \,{\left|\theta \right|}^{2}$$

where *y*_*i*_ is the actual category distribution, ${\hat{y}}_{i}$ is the predicted category distribution, and λ |θ|^2^ is the regular term.

## Experiment and Results Analysis

### Experimental Data Set and Evaluation Index

The dataset for the sentiment classification experiment uses four categories of products on the Jingdong website which have a large sales volume: Women’s Clothing (WC), Mobile Phones (MP), Refrigerator (R), and Travel Bags (TB). The dataset was annotated manually, and the review data sentiment classification was marked as positive or negative. Hold-out method is used to divide training set and test set, and it is common practice to use about 2/3∼4/5 samples for training. Therefore, the labeled data were divided into training and test sets in the ratio of 7:3. Each category includes 1,000 positive and 1,000 negative reviews, respectively, as shown in Table 2.

**TABLE 2:** Experimental data set.

Category	Positive reviews	Negative reviews
Women’s clothing (WC)	1000	1000
Mobile phones (MP)	1000	1000
Refrigerator (R)	1000	1000
Travel bags (TB)	1000	1000

**FIGURE 5:**
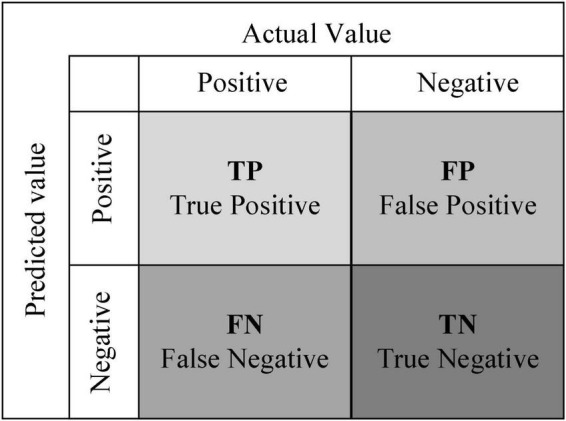
Confusion Matrix.

In evaluating the classification effectiveness of the sentiment classification model, the accuracy metric in the Confusion Matrix was used. The higher the accuracy rate, the better the classification effect of the model. The Confusion Matrix is shown in Figure 5.

The formula for calculating the accuracy is as follows:


(20) $$A\,c\,c\,u\,r\,a\,c\,y=\frac{\text{TP}+\text{TN}}{\text{TP}+\text{FP}+\text{TN}+\text{FN}}$$

### Parameter Setting

Python 7.2 and MATLAB 2016b software are used to implement the proposed model. In order to train a better model, it is important to set the model parameters, and the proposed CA-LSTM model parameters are set as shown in Table 3.

**TABLE 3:** Parameter setting.

Parameter description	Values
Word vector dimension	100
Batch size	64
Number of filters	100
Convolution kernel size	3
Dropout ratio	0.5
Optimization functions	Adam
Learning rate	0.001
Number of training iterations	20

### Classification Performance

**FIGURE 6:**
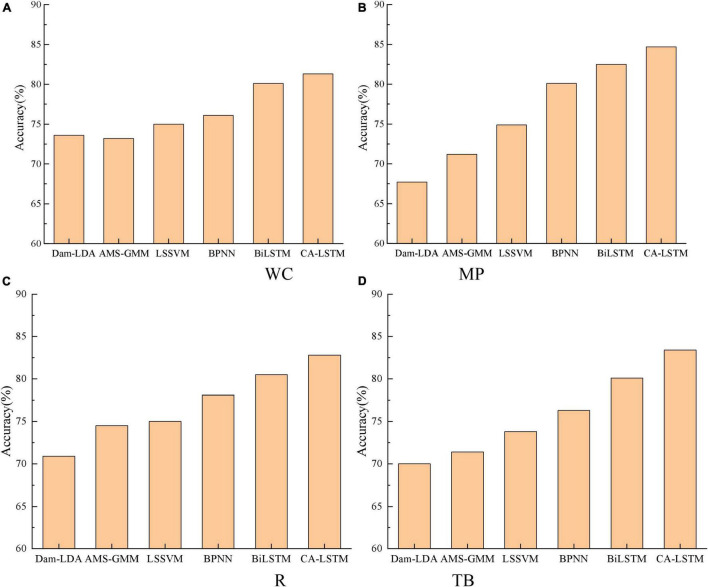
**(A)** Women’s Clothing (WC). **(B)** Mobile Phones (MP). **(C)** Refrigerator (R). **(D)** Travel Bags (TB). Accuracy comparison under different target domains.

To verify the effectiveness of the proposed CA-LSTM model in this paper, it is compared with Dam-LDA (Zhang et al., 2015), AMS-GMM (Zao et al., 2014), LSSVM (Liu, 2015), BPNN (Li et al., 2018), and BiLSTM (Contractor et al., 2021). The accuracy comparison results under different target domains are shown in Figure 6. The average accuracy of different methods on the experimental dataset is shown in Table 4.

**TABLE 4:** Average accuracy of different methods on the experimental dataset.

Classification model	Average accuracy (%)
Dam-LDA	73.1
AMS-GMM	74.8
LSSVM	76.2
BPNN	78.2
BiLSTM	81.5
CA-LSTM	83.3

As can be seen from Figure 6 and Table 4, the accuracy of the CA-LSTM model proposed in this paper is the highest in each sentiment analysis task, with an average accuracy of 83.3%. Compared with the Dam-LDA model, the average classification accuracy of the CA-LSTM model is improved by 14%. Compared with the AMS-GMM model, the average classification accuracy of the CA-LSTM model is improved by 11.3%. The average accuracy of the CA-LSTM model is improved by 9.3, 6.5, and 2.2% compared to the LSSVM, BPNN and BiLST models, respectively. This is because the CA-LSTM model makes full use of the spatial Boolean model to clarify the relationship between consumers’ real psychology and review attributes, thus helping to directly quantify the sentiment characteristics. In addition, the fusion of attention mechanism and convolutional operations into LSTM, thus allowing the extraction of real consumer sentiment feature indicators in the context of reviews, improves the correct rate of review text classification.

**FIGURE 7:**
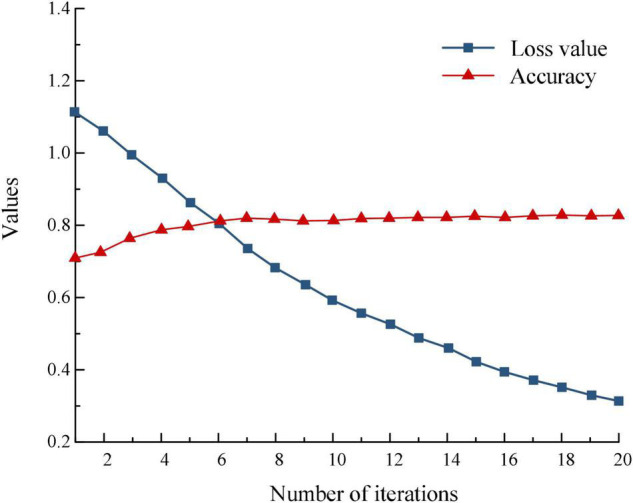
Accuracy and loss value variation curves.

Taking WC as an example, the curves of accuracy and loss values with increasing number of iterations are shown in Figure 7.

The experimental results show that with the increase in the number of selected generations, the accuracy rate as a whole shows an increasing trend eventually reaching a stable state, while the loss value shows a decreasing trend eventually also tends to be stable. Therefore, the classification accuracy of the proposed CA-LSTM model method can converge to the local optimal solution quickly, while there is a correlation between the loss value and the classification accuracy, and the larger the accuracy, the smaller the loss value. For four product categories, the proposed model can judge whether consumers’ emotions are positive or negative. There is little difference between women’s clothing, mobile phones, refrigerators and travel bags.

## Conclusion

In this paper, we propose a deep learning-based real online consumer sentiment classification model, CA-LSTM, which makes full use of a spatial Boolean model to specify the relationship between consumers’ real psychology and review attributes, thus helping to directly quantify the sentiment features. In addition, attention mechanisms and convolution operations are fused into the LSTM so that real consumer sentiment feature indicators can be extracted in the context of reviews. The experimental results show that the CA-LSTM model has the highest accuracy in each sentiment analysis task, with an average accuracy of 83.3%, which helps large e-commerce websites to accurately determine the true sentiment tendency in comments. However, since the CA-LSTM model must be retrained and then tested each time when dealing with real sentiment classification in the same target domain, cross-domain sentiment recognition cannot be achieved. Therefore, further work will be conducted in subsequent studies to address the migration of the models.

## Data Availability Statement

The raw data supporting the conclusions of this article will be made available by the authors, without undue reservation.

## Author Contributions

Both authors listed have made a substantial, direct and intellectual contribution to the work, and approved it for publication.

## Conflict of Interest

The authors declare that the research was conducted in the absence of any commercial or financial relationships that could be construed as a potential conflict of interest.

## Publisher’s Note

All claims expressed in this article are solely those of the authors and do not necessarily represent those of their affiliated organizations, or those of the publisher, the editors and the reviewers. Any product that may be evaluated in this article, or claim that may be made by its manufacturer, is not guaranteed or endorsed by the publisher.

## References

[B1] Alkubaisi G. A. A. J. Kamaruddin S. S. Husni H. (2018). Stock market classification model using sentiment analysis on twitter based on hybrid naive bayes classifiers. *Int. J. Eng. Technol.* 11 52–61. 10.5539/cis.v11n1p52

[B2] Cambria E. Poria S. Gelbukh A. Thelwall M. (2018). Sentiment analysis is a big suitcase. *Intell. Syst. IEEE* 32 74–80. 10.1109/MIS.2017.4531228

[B3] Capraro V. Vanzo A. (2018). Understanding moral preferences using sentiment analysis. *Soc. Sci. Electronic Publishing* 13 56–65. 10.2139/ssrn.3186134

[B4] Chen J. Hu B. Moore P. Zhang X. Ma X. (2015). Electroencephalogram-based emotion assessment system using ontology and data mining techniques. *Appl. Soft Comput.* 30 663–674. 10.1016/j.asoc.2015.01.007

[B5] Contractor D. Patra B. Mausam M. Singla P. (2021). Constrained bert bilstm crf for understanding multi-sentence entity-seeking questions. *Nat. Lang. Eng.* 27 65–87. 10.1017/S1351324920000017

[B6] Dehkharghani R. Mercan H. Javeed A. Saygin Y. (2014). Sentimental causal rule discovery from twitter. *Expert Syst. Appl.* 41 4950–4958. 10.1016/j.eswa.2014.02.024

[B7] Dragoni M. Soujanya P. Cambria E. (2018). Ontosenticnet: a commonsense ontology for sentiment analysis. *IEEE Intell. Syst.* 55 115–124. 10.1109/MIS.2018.033001419

[B8] Ghiassi M. Lee S. (2018). A domain transferable lexicon set for twitter sentiment analysis using a supervised machine learning approach. *Expert Syst. Appl.* 106 197–216. 10.1016/j.eswa.2018.04.006

[B9] Hou Y. (2020). Students’ emotional analysis on ideological and political teaching classes based on artificial intelligence and data mining. *J. Intell. Fuzzy Syst.* 40 1–9. 10.3233/JIFS-189413

[B10] Kakad H. Dhage S. (2021). Cross domain-based ontology construction via jaccard semantic similarity with hybrid optimization model. *Expert Syst. Appl.* 178 115–123. 10.1016/j.eswa.2021.115046

[B11] Kang Q. Lei S. Zhou M. Wang X. Wei Z. (2018). A distance-based weighted undersampling scheme for support vector machines and its application to imbalanced classification. *IEEE Trans. Neural Netw. Learn. Syst.* 29 4152–4165. 10.1109/TNNLS.2017.2755595 29990027

[B12] Khan A. Li J. P. Haq A. U. Memon I. Din S. U. (2020). Emotional-physic analysis using multi-feature hybrid classification. *J. Intell. Fuzzy Syst.* 40 1–14. 10.3233/JIFS-201069

[B13] Lanjewar R. B. Mathurkar S. Patel N. (2015). Implementation and comparison of speech emotion recognition system using gaussian mixture model (gmm) and k- nearest neighbor (k-nn) techniques. *Procedia Comput. Sci.* 49 50–57. 10.1016/j.procs.2015.04.226

[B14] Li S. Chen T. Wang L. Ming C. (2018). Effective tourist volume forecasting supported by pca and improved bpnn using baidu index. *Tour. Manag.* 68 116–126. 10.1016/j.tourman.2018.03.006

[B15] Li Z. Li Q. Zou X. Ren J. (2021). Causality extraction based on self-attentive bilstm-crf with transferred embeddings - sciencedirect. *Neurocomputing* 423 207–219. 10.1016/j.neucom.2020.08.078

[B16] Liu K. Zhang J. (2021). A dual-layer attention-based lstm network for fed-batch fermentation process modelling - sciencedirect. *Comput. Aided Chem. Eng.* 50 541–547. 10.1016/B978-0-323-88506-5.50086-3

[B17] Liu P. Zhang L. Gulla J. A. (2021). Multilingual review-aware deep recommender system via aspect-based sentiment analysis. *ACM Trans. Inform. Syst.* 39 1–33. 10.1145/3432049

[B18] Liu Z. (2015). Research for public opinion of charitable organizations based on microblogging sentiment analysis. *J. Inform. Comput. Sci.* 12 1011–1019. 10.12733/jics20105433

[B19] Mäntylä M. V. Graziotin D. Kuutila M. (2018). The evolution of sentiment analysis—a review of research topics, venues, and top cited papers. *Comput. Sci. Rev.* 27 16–32. 10.1016/j.cosrev.2017.10.002

[B20] Naeem S. Mashwani W. K. Ali A. Uddin M. I. Khan N. A. (2021). Machine learning-based usd/pkr exchange rate forecasting using sentiment analysis of twitter data. *Comput. Mater. Contin.* 67 3451–3461. 10.32604/cmc.2021.015872

[B21] Rehman A. Jaafar J. Omar M. Basri S. Hilmi M. (2017). Suitable personality traits for learning programming subjects: a rough-fuzzy model. *Int. J. Adv. Comput. Sci. Appl.* 8 153–162. 10.14569/IJACSA.2017.080820

[B22] Samah E. A. K. A. (2021). Nave bayes twitter sentiment analysis in visualizing the reputation of communication service providers: during covid-19 pandemic. *Turk. J. Comput. Math. Educ.* 12 1753–1764. 10.17762/turcomat.v12i5.2176

[B23] Shamrat F. M. J. M. Chakraborty S. Imran M. M. Muna J. N. Rahman M. O. (2021). Sentiment analysis on twitter tweets about covid-19 vaccines using nlp and supervised knn classification algorithm. *Indones. J. Electr. Eng. Comput. Sci.* 23 463–470. 10.11591/ijeecs.v23.i1.pp463-470

[B24] Suhasini M. Badugu S. (2018). Two step approach for emotion detection on twitter data. *Int. J. Comput. Appl.* 179 12–19. 10.5120/ijca2018917350 7911558

[B25] Tao W. Liu T. (2018). Building ontology for different emotional contexts and multilingual environment in opinion mining. *Intelligent Automation and Soft Computing* 24 65–71. 10.1080/10798587.2016.1267243

[B26] Wei Z. Guan Z. Long C. He X. Deng C. Wang B. (2018). Weakly-supervised deep embedding for product review sentiment analysis. *IEEE Trans. Knowl. Data Eng.* 30 185–197. 10.1109/TKDE.2017.2756658

[B27] Weng B. Lin L. Xing W. Megahed F. M. Waldyn M. (2018). Predicting short-term stock prices using ensemble methods and online data sources. *Expert Syst. Appl.* 112 258–273. 10.1016/j.eswa.2018.06.016

[B28] Zao L. Cavalcante D. Rui C. (2014). Time-frequency feature and ams-gmm mask for acoustic emotion classification. *IEEE Signal Process. Lett.* 21 620–624. 10.1109/LSP.2014.2311435

[B29] Zhang S. Shi C. Jiang X. Zhang Y. Zhang L. (2020). Analysis of the trend of global power sources based on comment emotion mining. *Global Energy Interconnection* 3 283–291. 10.1016/j.gloei.2020.07.009

[B30] Zhang X. Zha C. Xu X. Song P. Zhao L. (2015). Speech emotion recognition based on lda ++ kernel-knnflc. *Dongnan Daxue Xuebao (Ziran Kexue Ban)/J. Southeast Univer. (Nat. Sci. Ed.)* 45 5–11. 10.3969/j.issn.1001-0505.2015.01.002

